# Developmental Associations between Cognition and Adaptive Behavior in Intellectual and Developmental Disability

**DOI:** 10.21203/rs.3.rs-3684708/v1

**Published:** 2024-01-08

**Authors:** Andrew Dakopolos, Emma Condy, Elizabeth Smith, Danielle Harvey, Aaron J Kaat, Jeanine Coleman, Karen Riley, Elizabeth Berry-Kravis, David Hessl

**Affiliations:** University of California Davis MIND Institute; Hofstra University; Cincinnati Children’s Hospital Medical Center Burnet Campus: Cincinnati Children’s Hospital Medical Center; University of California Davis; Northwestern University Feinberg School of Medicine; Regis University; Slippery Rock University of Pennsylvania; Rush University Medical Center; UC Davis MIND Institute

**Keywords:** cognition, intellectual and developmental disability, NIH Toolbox, fragile X syndrome, Down syndrome, adaptive behavior, latent change, structural equation modeling, longitudinal studies

## Abstract

**Background.:**

Intellectual and developmental disabilities (IDDs) are associated with both cognitive challenges and difficulties in conceptual, social, and practical areas of living (DSM–5). Individuals with IDD often present with an intellectual disability in addition to a developmental disability such as autism or Down syndrome. Those with IDD may present with deficits in intellectual functioning as well as adaptive functioning that interfere with independence and living skills. The present study sought to examine associations of longitudinal developmental change in domains of cognition (NIH Toolbox Cognition Battery, NIHTB-CB) and adaptive behavior domains (Vineland Adaptive Behavior Scales-3; VABS-3) including Socialization, Communication, and Daily Living Skills (DLS) over a two-year period.

**Methods.:**

Eligible participants for this multisite longitudinal study included those who were between 6 and 26 years at Visit 1, and who had a diagnosis of, or suspected intellectual disability (ID), including borderline ID. Three groups were recruited, including those with fragile X syndrome, Down syndrome, and other/idiopathic intellectual disability. In order to examine the association of developmental change between cognitive and adaptive behavior domains, bivariate latent change score (BLCS) models were fit to compare change in the three cognitive domains measured by the NIHTB-CB (Fluid, Crystallized, Composite) and the three adaptive behavior domains measured by the VABS-3 (Communication, DLS, and Socialization).

**Results.:**

Over a two-year period, change in cognition (both Crystalized and Composite) was significantly and positively associated with change in daily living skills. Also, baseline cognition level predicted growth in adaptive behavior, however baseline adaptive behavior did not predict growth in cognition in any model.

**Conclusions.:**

The present study demonstrated that developmental improvements in cognition and adaptive behavior are associated in children and young adults with IDD, indicating the potential for cross-domain effects of intervention. Notably, improvements in Daily Living Skills on the VABS-3 emerged as a primary area of adaptive behavior that positively related to improvements in cognition. This work provides evidence for the clinical, “real life” meaningfulness of the NIHTB-CB in IDD, and important empirical support for the NIHTB-CB as a fit-for-purpose performance-based outcome measure for this population.

## Background

Intellectual and developmental disabilities (IDDs) are associated with both cognitive challenges and difficulties in conceptual, social, and practical areas of living (DSM–5^1^). Those with IDD typically present with deficits in intellectual functioning including reasoning, problem solving, planning, abstract thinking, judgment, academic learning, and learning from experience, as well as deficits in adaptive functioning that interfere with independence and living skills within the context of social and cultural developmental norms and expectations^1^. Adaptive functioning refers to one’s ability to function independently across home and community contexts throughout the lifespan^2^ and in many individuals with IDD, is an important measure of well-being both at a given moment^3,4^ and over time^5,6^. There is evidence that adaptive behaviors may also serve as a better marker of overall functioning within one’s environment than intellectual ability alone for this population^3,4^.

There is abundant and well characterized cross-sectional^7,8^ and longitudinal^9–12^ evidence of deficits in cognition^13–17^ and adaptive behavior^9,18–20^ among specific etiologies within IDD (i.e., fragile X syndrome [FXS], Down syndrome [DS], and Williams syndrome [WS]) in development. This strong foundation in the literature has identified phenotypic patterns of relative strengths and weaknesses in both cognitive and adaptive domains across IDD etiologies over time. However, less is known about how cognition and adaptive behavior develop *together* in individuals with IDD. Some have assessed *both* adaptive behavior and cognition longitudinally, *but separately*. For example, in a sample of children aged 3–12 years with autism spectrum disorder compared to those with FXS, differential patterns of longitudinal change were observed in both intelligence and adaptive behavior across groups, yet no direct associations of longitudinal change were made *between* domains of cognitive and adaptive function^21,22^, which can limit a full understanding of the ways in which specific aspects of cognition may impact certain domains of daily functioning. Also, recently the validity of these results and others has been more closely scrutinized in terms of the profile and performance of scores (e.g., standard scores, raw scores, age-equivalent scores) used in analyses^23^. These critiques center around issues of floor-effects and scaling of scores for individuals – often those with IDD – who perform near or below the floor, and whether standard scores or age-adjusted scores adequately reflect individuals’ performance in such cases.

In another study, 58 adults with DS (ages 31–55 years) were seen up to seven times over a 10-year period, and change in both adaptive behavior (using the Inventory for Client and Agency Planning) and cognitive skills (using the Woodcock-Johnson Early Developmental Battery) was assessed^24^. Results revealed a modest decline in all areas of adaptive function (i.e., social communication, community living, motor skills, personal living skills, broad independence) over time, however a differential pattern of growth and decline emerged for cognitive skills. Comprehension, knowledge, and auditory processing continued to improve into the seventh decade of life, however measures of short-term memory, long-term memory, and visual processing revealed maximum performance around 50 years, followed by decline in these skills^24^. Again, associations between adaptive behavior and cognitive skills were not made directly, yet these results indicate that in adulthood, specific areas of cognitive and adaptive functioning may track together in people with DS^24^.

More recently, Hahn et al., (2015) assessed the effect of non-verbal cognition (measured by the Mullen Scales of Early Learning taken at time 1) on the rate of growth (slopes) and starting level (intercepts) of adaptive behavior subscales in children with FXS. They found that non-verbal cognition significantly predicted the rate of growth in daily living skills and motor domains, and significantly predicted the starting level in socialization, communication, and motor domains^11^. In a similar study, intelligence (Using the Kaufman Brief Intelligence Test, 2nd Edition) and adaptive behavior (using the Vineland Adaptive Behavior Scales, Second Edition) were examined longitudinally in a sample of children with WS between the ages of 14 and 49 years. Intelligence remained stable while adaptive behavior decreased over time^10^. Baseline intelligence was correlated with a higher intercept (i.e., starting level ) of adaptive behavior across all domains (i.e., communication, daily living, socialization), but higher baseline intelligence also predicted greater decreases in adaptive behavior composite and communication scores over time^10^.

To our knowledge, no study to-date has investigated the association *between* developmental changes in cognitive skills and adaptive behavior in children or adults with IDD. It is important to characterize how these two broad domains track *within* people with IDD over time as they are the primary areas of deficit in this population across the lifespan. Moreover, understanding both the direction and patterns of association between adaptive behavior and cognition is especially relevant to educational, behavioral, and pharmacological interventions. Specifically, there is great utility in investigating whether improvements in particular cognitive skills – measured by performance-based tests – that may be targeted in treatment, track with clinically meaningful improvements in adaptive behavior. As cognitive tests are increasingly utilized as key performance-based clinical outcome assessments (what the Food and Drug Administration [FDA] refer to as a “PerfO”^25^) in clinical trials and other treatments for people with IDD, it is critical to understand how changes in cognition as measured by such instruments may associate with and perhaps impact adaptive or functional changes in the individual’s daily life.

Although there is no cure or significant disease modifying treatment for any form of IDD, there have been promising results in animal models, and strong translational research based in FXS and DS for targeted treatments^15,26–28^. Human trials utilizing various classes of medications in both FXS (see Berry-Kravis et al., 2018; for review) and DS^29–32^ have not been met with the same successes as these treatments in animal models, possibly due to challenges in translation from animal models to humans, maintaining safe and adequate dosages, or inappropriate, insensitive, or invalid outcome measures^27,33^. Despite these setbacks, recent studies have made adjustments to outcome measures that have led to exciting advances. In a double-blind, placebo-controlled trial, adults with DS made significant improvements in recognition memory, inhibitory control, and caregiver-reported functional academics over a one-year period when treated with green tea extract containing epigallocatechin-3-gallate^30^. There is also evidence that components of the NIH Toolbox Cognition Battery (NIHTB-CB)^34^ detected treatment effects in a 24-week phase 2 randomized, placebo-controlled, crossover trial of a phosphodiesterase-4D allosteric inhibitor (BPN14770) in 30 adult males with FXS^35^. In this study, compared to placebo, cognitive improvement in BPN14470-treated patients was detected by the language-based NIHTB-CB Crystallized Cognition Composite score (comprised of the Oral Reading Recognition and Picture Vocabulary tests).

Prospective clinical trials continue to build upon this important work; however, a critical question remains unanswered: do improvements made in cognitive skills, as measured by such tests, *extend to adaptive improvements* in the everyday lives of people with ID? Though newly deployed outcome measures such as the NIHTB-CB have demonstrated reliability, validity, and treatment sensitivity in a clinical trial^35^, it is imperative that we determine whether different degrees of improvement on clinic-based cognitive tests are associated with changes in the daily functioning of people with IDD. Whether or not improvements in outcomes (i.e., cognitive skills from the NIHTB-CB) translate into identifiable and clinically significant improvements in downstream areas of functioning (such as academic skills, activities of daily living, communication, or social skills) will likely be a key determinant for clinical trials moving forward, and ultimate FDA acceptance of key outcome measures and eventual approval of targeted medications.

Our psychometric studies of the NIHTB-CB in children, adolescents, and young adults with IDD have demonstrated its feasibility and validity^7,36^, as well as its sensitivity to developmental change^12^ among this population. Therefore, the present study sought to build upon this previous work with a data-driven approach, to explore associations of longitudinal change in domains of cognition (i.e., NIHTB-CB subtests) and adaptive behavior domains (i.e., VABS-3 Socialization, Communication and Daily Living Skills) over a two-year period. Our previous work has identified some limitations using the NIHTB-CB in individuals with IDDs, particularly for those with lower mental ages (i.e., < 5 years)^7^. A specific limitation relates to composite scores in the NIHTB-CB. For instance, the Fluid Cognition Composite is comprised of five subtests, all of which must be administered, valid, and have a “completed” status in order to produce the composite score. Many individuals in our longitudinal sample are unable to pass practice and thus complete all five subtests. Therefore, they do not have composite scores available, thus limiting our power to test hypotheses at the construct level. Structural equation modeling (SEM) provides an analytic framework to help combat this particular issue, given that this modeling approach is robust to missing data, aiding our ability to retain the full sample in our models – even if a single participant was able to provide only one valid NIHTB-CB subtest score. In the present study, bivariate latent change score (BLCS) models provided two-year estimates of both cognitive and adaptive behavior change in individuals with FXS, DS, and other intellectual disability (OID). This modeling framework allowed us to examine the association between latent change for cognition and adaptive behavior across construct levels of each assessment.

## Methods

### Participants

Eligible participants for this multisite longitudinal study included those who were between 6 and 26 years at Visit 1, and who had a diagnosis of, or suspected IDD. During Visit 1, ID or borderline ID criteria were based on the DSM-5^1^, with adaptive behavior deficits measured by the Vineland Adaptive Behavior Scales, Third Edition (VABS-3)^2^ and IQ < 80 on the Stanford-Binet Intelligence Scales, 5th Edition (SB5). Three groups were recruited: FXS (full mutation, with genetic confirmation), DS (with genetic confirmation if possible), and OID (with genetic confirmation of negative fragile X mutation). A mental age equivalent of at least 3.0 years as measured by the SB5 was required, in concordance with NIHTB-CB age limits. Participants were required to be stable with usual treatment for at least 4 weeks before each visit. Exclusion criteria consisted of uncorrectable or uncorrected vision impairment, significant motor impairment preventing touch screen or keypad responses, or history of head injury, brain infection, stroke, or other neurological problems such as uncontrolled daily seizures or excessive sedation from medication. Recruitment sources consisted of research registries, flyers at local clinics, announcements through parent support foundation websites, and mailings to families registered with state departments that provide services to individuals with IDD. A total of 318 participants with IDD were recruited at Visit 1, and of those recruited, 54 individuals were ineligible: 20 with IQ > 79 and 34 with mental age below 3 years, leaving a final sample of 264. Full protocol, details of the NIHTB-CB, and its performance at baseline in the present ID samples has been reported previously^7,12,36^.

### Protocol

The NIHTB-CB, VABS-3 interview and SB5 were completed at Visit 1. For some participants, assessments were conducted over two days. After completion of the SB5, participants completed the NIHTB-CB while their parent/caregiver completed the VABS-3 with a psychologist or trained personnel. The same procedure was conducted again approximately 2-years later at Visit 2.

### Measures

The NIHTB-CB^37^ is an iPad-based assessment that provides information about fluid cognition, crystalized cognition, and a cognition composite through 7 tests. Flanker Inhibitory Control and Attention (FICA), Dimensional Change Card Sort (DCCS), List Sorting Working Memory (LSWM), Pattern Comparison Processing Speed (PCPS), and Picture Sequence Memory (PSM) comprise the Fluid Cognition Composite, and Picture Vocabulary (PV) and Oral Reading Recognition (ORR) comprise the Crystalized Cognition Composite. The two composites combine for the Total Cognition Composite score. A published manual of standardized NIHTB-CB administration procedures for IDD can be found in Ref.^38^. In the present study, the NIHTB-CB Version 2.0 was used. Unadjusted standard scores (USSs; non-age adjusted standard scores) were used for all Toolbox tests. USSs have a mean of 100 and SD of 15. The USSs are recommended for longitudinal measurement because, like change sensitive or growth scale scores, they are not adjusted based on age-related growth of normative peers.

The Vineland Adaptive Behavior Scales 3rd ed. (VABS-3)^2^ interview form was used to measure adaptive behavior (AB) domains including Communication (consisting of Expressive Language, Receptive Language, and Written Language), Daily Living Skills (DLS; consisting of Personal, Home, and Community skills), and Socialization (consisting of Interpersonal Relationships, Play and Leisure, and Coping Skills). For the present study, VABS-3 growth scale values (GSVs) were used for all analyses as they have been shown to be sensitive in individuals with IDD, particularly given their robust performance longitudinally and being less susceptible to floor effects^39,40^.

The Stanford-Binet Intelligence Scales, 5th ed. (SB5), which is standardized for individuals between 2–85 years, provides an overall index of intellectual ability reported as the Full-Scale IQ (FSIQ). In part, due to its broad developmental range, the SB5 has performed well in our prior studies of IDD ^7,36,41^. Our protocol utilizes mental (rather than chronologic) age to select NIHTB-CB test versions and VABS-3 start points, which were derived from the SB5 FSIQ^38^ for each participant.

### Statistical Analyses

In order to examine the association of developmental change between cognitive and adaptive behavior domains, permutations of bivariate latent change score (BLCS) models were used to compare change in the three cognitive domains measured by the NIHTB-CB (Fluid, Crystallized, Composite) and the three AB domains measured by the VABS-3 (Communication, DLS, and Socialization), resulting in the evaluation of nine models plus one full model (including all cognitive domains and all AB domains). Latent change score models are a type of structural equation modeling that provide estimates of change as latent variables based on two or more time points. In the BLCS framework, each model can assess the association between the latent change estimated for two constructs of interest^42^. We have utilized latent change scores previously to characterize developmental change in this sample across individual NIHTB-CB subtests^12^. Missing data were handled with full information maximum likelihood estimation, which is a standard recommendation to provide accurate parameter estimates in the presence of missing data^43^.

Generally, each model contained latent scores for cognition and AB at Visit 1 and Visit 2 that were derived from the observed scores from each domain’s respective subtests^42,44–47^. Latent change scores for cognition (ΔCognition) and AB (ΔAB) were included to model change from Visit 1 to Visit 2. Furthermore, we included an estimate of the correlated change between ΔAB and ΔCognition in each model to assess cross-domain coupling of adaptive behavior and cognition. Time between visits and participant age were each used as a covariates at the latent level in all models to control for any differences in cognitive and AB change due to variations in timing between Visit 1 and Visit 2, as well as age-related changes – modeled as those between 6 and 16 years at Visit 1, and those 16 years or older at Visit 1, which we have previously demonstrated in this population^12^. Analyses included all participants with a valid NIHTB-CB score, even without completion of visit 2 as BLCS models are robust to missingness^42^. Supplementary Fig. 1 graphically presents a generic representation of these models, and Table 3 provides details of each model’s specification. For model fit we first specified base models in which nothing was correlated, and each variable received an equated intercept and variance across time^48^. We then assessed each model’s fit by comparing to its base model (utilizing robust fit parameters including CFI, TLI, and RMSEA) using methods from Savalei^49^, and indices of fit based on Little^50^ (i.e., CFI > 0.85; TLI > 0.85; RMSEA < .08).

## Results

### Descriptive statistics

A sample of 264 individuals were included in the present analyses. Descriptive statistics for sex assigned at birth, race, ethnicity, and diagnostic group are provided in Table 1 and for cognitive and adaptive behavior scores at Visit 1 in Table 2.

### Bivariate latent change score model fit evaluation

Twelve bivariate latent change score models were conducted assessing the relationship between change in the adaptive behavior (ΔAB) domains and change in the cognition (ΔCOG) domains from Visit 1 to Visit 2. The interval between Visit 1 and Visit 2 in years (*M* = 2.44, *SD* = 0.81) was included as a covariate at the latent level, and age, split into those between 6 and 16 years, and 16 years or older at Visit 1 was included as a covariate at the Visit 1 level, as well as the latent level. A model fit statistic summary is presented in Table 3.

The first six models (Models 1–6) assessed ΔAB, where AB was modeled as one of three VABS-3 subscales: Communication (Comm.), Socialization (Soc.), and Daily Living Skills (DLS), and ΔCOG, where COG was modeled as one of two NIHTB-CB composites (Fluid and Crystallized). Of these models, Model 1 and Model 2 demonstrated relatively poor model fit. These Models were followed up with Models 1.1 and 2.1, which omitted written communication from the AB Communication domain, and were subsequently found to have good fit. Model 6 did not demonstrate adequate model fit, and was not further evaluated. Models 3, 4, and 5 were deemed to have good fit.

**Figure 1:**
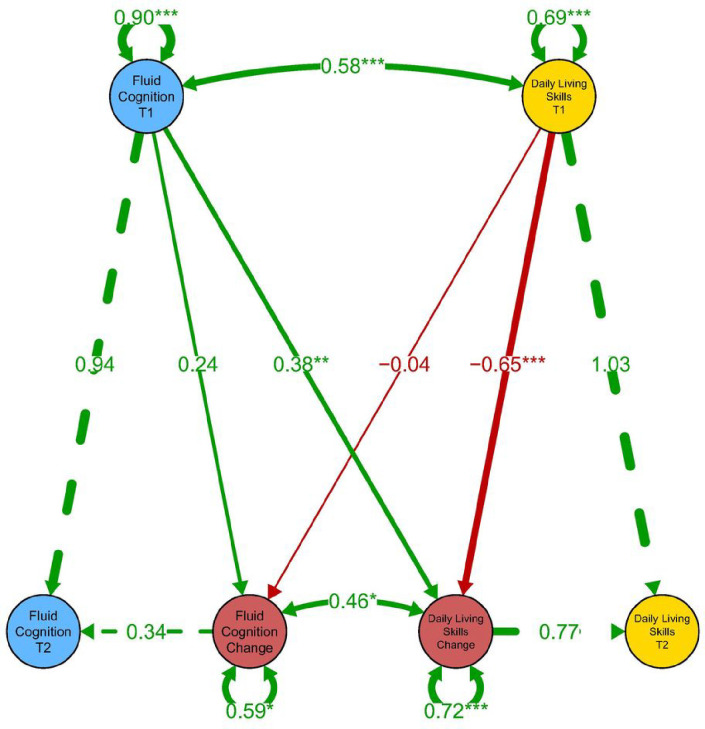
Structural equation model diagram of representative Model B showing association between latent constructs of cognition (COG; NIH Toolbox Cognition Battery) and adaptive behavior (AB; Vineland-3 Daily Living Skills) at Visits 1 and 2 and latent change of these constructs across 2 years of development in youth with IDD. Manifest variables omitted for visual clarity. Raw solution with standardized solution in parenthesis.

The next set of models (Models A-C) assessed ΔAB across the three subscales of the VABS-3 (Comm., Soc., and DLS) and ΔCOG across the full NIHTB-CB (comprised of its seven subtests). Of these models, Models B and C were deemed to have acceptable fit. See Fig. 1 for a graphical representation of the SEM for Model B.

A final model (Full Model) assessed ΔAB across the VABS-3 (all domains) and ΔCOG across the full NIHTB-CB. The model fit was poor and not examined further. Notably, none of the models where AB was modeled using the VABS-3 Comm. subscales were shown to have good fit until removing the written communication subdomain. However, for the other two AB domains (DLS & Soc.), models wherein COG was defined as either Crystallized, Fluid, or the full NIHTB-CB were shown to have good fit.

### Correlation between cognitive and adaptive behavior change

**Figure 2:**
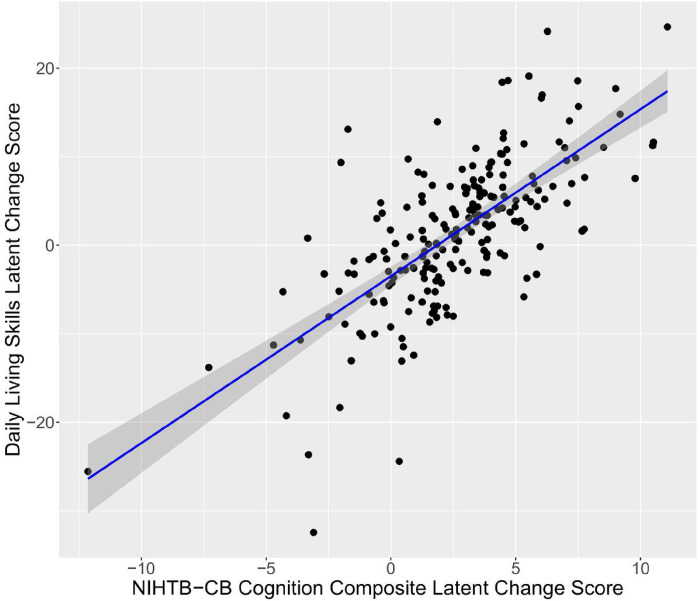
Linear association (with SE shaded) between latent change scores for VABS-3 Daily Living Skills (y-axis) and NIHTB-CB Cognition Composite (x-axis) for Model B.

In two of the seven models of good fit, a significant relationship was observed between the change in cognition (ΔCOG) and change in adaptive behavior (ΔAB). A positive relationship between the ΔCOG and ΔAB was observed in Model 3, where COG was a variable comprised of the NIHTB-CB subscales in the Crystallized domain (PV and ORR) and AB was comprised of the VABS-3 subscales in the DLS domain (Personal, Domestic, and Community). Similarly, a positive relationship between ΔCOG and ΔAB was observed in Model B, where AB was again comprised of the VABS-3 subscales in the DLS domain, but COG was comprised of all of the NIHTB-CB subscales (Fig. 2). These findings indicate that change in cognition, specifically in the Crystallized domain, relates to change in daily living skills over time in our sample. A summary of the parameters of interest from these models is provided in Table 4.

### Visit 1 Cross-domain coupling of AB and COG

Tables 5 and 6 present regression parameters for COG at Visit 1, and AB at Visit 1 respectively predicting ΔCOG and ΔAB. Regression components of the models with good fit (i.e., Models 3, 4, 5, B, C, 1.1 and 2.1) were evaluated to examine the influence of COG at Visit 1 on ΔAB and ΔCOG (Table 5), and AB at Visit 1 on ΔAB and ΔCOG (Table 6).

COG at Visit 1 significantly predicted increased developmental change in AB for Models 4, and 2.1, as well as Models B and C, however COG at Visit 1 did *not* predict ΔCOG in any model. This pattern of results indicates that both Crystalized Cognition, Fluid Cognition and the Total Cognition Composites are good indicators of developmental change in Daily Living Skills, Socialization, and expressive/receptive language; however individual starting cognition scores are not predictive of individuals’ subsequent cognitive development.

For the cross-domain coupling of AB at Visit 1 on ΔCOG, AB at Time 1 did *not* predict ΔCOG in any model. AB at Visit 1 predicted less change in ΔAB for all models (i.e., Models 3, 4, 5, 1.1, B, and C) indicating that those with higher DLS and Socialization, and expressive/receptive communication scores at Visit 1 reported less improvement in those respective adaptive behavior skills after two years.

## Discussion

The present study sought to examine the relationship between developmental change in cognition and adaptive behavior in children and young adults with IDD. We modeled this association using bivariate latent change score models. We found that developmental improvements in language-based crystalized cognition as measured by the NIHTB-CB were related to improvement in daily living skills, and that improvement in overall cognition was also related to improvements in daily living skills. Models that included the VABS-3 Communication domain (i.e., Models 1, 2, A, and the Full Model) did not have adequate model fit for analysis. Follow-up analyses indicated that the measurement model for VABS-3 Communication did not fit, with the written communication domain demonstrating a high degree of covariance with the other areas comprising VABS-3 Communication (i.e., receptive communication and expressive communication). Models excluding written communication were subsequently fit (Model 1.1, 2.1).

The present study is the first, to our knowledge, to assess the relation between developmental change in cognition and adaptive behavior in individuals with IDD, two particularly important areas of functioning for this population, as deficits in each of these areas constitute core featiures of IDD. The present study demonstrates that developmental improvements in cognition and adaptive behavior are associated in children and young adults with IDD, indicating the potential for cross-domain effects of intervention. Notably, improvements in Daily Living Skills on the VABS-3 emerged as a primary area of adaptive behavior that positively related to improvements in cognition. Developmental change in all domains of adaptive behavior were predicted by cognitive skills at Visit 1; specifically Fluid Cognition at Visit 1 predicted improvements in DLS and Communication, and the Cognition Composite at Visit 1 predicted improvement in DLS and Socialization.

These findings demonstrate some of the “real-life” improvements that are associated with cognitive growth in a longitudinal study of youth with IDD. Previous work has shown that cognitive ability, as measured via IQ testing, is correlated with adaptive functioning^51^ at the population level. With the strength of this relationship in mind, it begs the question whether changes in one of these domains will result in change in the other, particularly in individuals with lower IQ. Establishing such reciprocity has implications for how treatment trials in IDD are designed and implemented. However, until now, no previous studies have looked at how change in these abilities over time might relate to one another in IDD; rather, only limited work characterizing the natural history of cognitive ability and/or adaptive functioning in IDD with specific etiologies, such as WS^10^, FXS^23^ and DS^27^. Our findings indicate that changes in cognitive ability, as measured via the NIHTB-CB, are related to changes in adaptive functioning, as measured by the VABS-3, over only a two-year period.

In addition to the relationship between changes in cognitive ability and changes in adaptive behavior, an individual’s ability in these domains at the first visit also predicted change within and between these domains. This was particularly evident within the adaptive behavior domain, as starting with higher adaptive behavior skills at the first visit was associated with less growth in adaptive behavior over the following 2 years in all models. Such a pattern could be indicative of a regression to the mean, or evidence that as an individual approaches a skill ceiling, their growth in that domain will begin to slow. Interestingly, this effect was not apparent within the cognitive domain, as cognitive ability at Visit 1 was not significantly associated with change in cognitive ability, only with change in adaptive behavior. The models revealed a positive relationship such that higher cognitive ability at Visit 1 was related to increased growth in adaptive behavior. Taken with the cross-domain findings indicating that changes in cognition were associated with changes in adaptive behavior, these findings indicate that targeting improvement in cognitive skills in ID may result in positive adaptive behavior change. However, the present study cannot draw any causal inferences as it was correlational. Future experiments could verify whether changes in one domain cause change in the other, and innovative cognitive interventions studied experimentally with controlled, randomized clinical trials^52,53^, examining the impact on adaptive behaviors, could be fruitful.

The importance of developing endpoint measures in the field of ID is evident,^54^ with many existing measures of the concept of interest (i.e., cognition) deemed inadequate or not fully “fit for purpose”. Measures commonly used in clinical trials have been critiqued for their limitations in validation and sensitivity to change in individuals with IDD^55^. When evaluating clinical outcome assessments (COAs) for ID, PerfO’s (i.e., direct assessments) of cognition are limited because they largely assess general cognition (e.g., IQ tests) and are less likely to show short-term change; thus, COAs for ID often consist of observer reports or clinician reported outcomes^56^. The NIHTB-CB was developed, in part, with the express purpose of filling the performance outcome gap for cognition in intervention studies. However, it was not created, validated, or normed with consideration of IDD, a population currently undergoing clinical trials targeting cognition and in urgent need of suitable primary outcome measures. Nonetheless, validity evidence for the NIHTB-CB has been collected in IDD^7^ and it shows sensitivity to change in this population^12^. Evaluating the clinical meaningfulness of the NIHTB-CB was an important next step. The present study established its clinical meaningfulness through its relation to adaptive behaviors. The VABS-3 is often used as an outcome measure in clinical trials for ID; however, the VABS-3 is not a direct assessment (it is a combination of an observer report outcome and clinician-reported outcome), perhaps limiting its sensitivity, nor does it measure the concept often being targeted in many current or planned clinical trials for IDD (e.g., cognition). The NIHTB-CB remedies these concerns, and based on the findings of the present study, also characterizes change that relates to established clinically meaningful outcomes, such as adaptive behavior.

Latent change score models were used to resolve issues with missing data and the use of growth scale values (GSVs) in the present study. Missing data were particularly problematic for NIHTB-CB tests in the Fluid domain, notably Flanker and DCCS. These tasks are challenging for individuals with IDD^7^ as well as individuals of young mental ages, including young typically developing children^57^. Unfortunately, missing scores on any individual NIHTB-CB test prohibits the generation of Fluid, Crystallized, and Composite scores. For this reason, large portions of our sample would have been excluded from the analyses if a latent variable approach had not been used to model the cognitive domains. Additionally, VABS-3 GSVs are only available at the subdomain level. GSVs cannot be averaged across subdomains to create composites for Communication, Socialization, and DLS domains because they are “a unitless measure and therefore cannot be compared or combined across subdomains”^58^. By using latent change score models, we were able to use our data in full to examine the broader constructs of cognition and adaptive functioning. However, this approach presents practical challenges. Namely, the latent variable “scores” in these models thus do not match the composite or domain scores generated by the NIHTB-CB or the VABS-3. For this reason, the relationships between latent variables cannot be translated into practical terms regarding the standard output that are generated by these tests (e.g., “An *x* point change in the NIHTB-CB Crystallized Composite is associated with a y point change in the VABS-3 ABC). The present study instead provides evidence that change in certain domains of cognitive function and adaptive behavior are related at the latent level. These findings provide direction for future work at the measurement level as the domains of cognition and adaptive behavior can potentially be more narrowly specified in future studies. Another study caveat to emphasize pertains to the time span between assessments (approximately two years), and the number of observations across development (maximum of two), factors that likely reduced power to detect associations of cognition and adaptive behavior growth. More observations over a longer period of development would likely produce better and stronger estimates of these associations.

Related to the data missingness of the NIHTB-CB tests, future development of the NIHTB-CB should involve individuals with IDD to improve the probability that the measure can be used in clinical trials targeting cognition with this population. The development of the NIH Infant and Toddler (Baby) Toolbox (NBT), including domains of cognition and executive function, language, numeracy/early mathematics, motor, and social functioning, is currently underway. The NBT aims to capture neurodevelopment at younger ages (1–42 months old) for both research and clinical use. Individuals with IDD represent a clear clinical population of interest for this measure, particularly due to the much lower mental ages often seen in this population, and the limited feasibility we have observed for some fluid reasoning tests in individuals with these lower mental ages.

## Conclusions

In summary, the present study demonstrated that cognitive level, as well as change in cognition over a two-year period of development, as measured by the NIHTB-CB, are associated with growth in adaptive behavior, especially daily living skills, among youth with intellectual and developmental disabilities. This work provides evidence for the clinical, “real life” meaningfulness of the NIHTB-CB in IDD, and important empirical support for the NIHTB-CB as a fit-for-purpose performance-based outcome measure for this population.

## Figures and Tables

**Table 1:** Descriptive statistics for participants

Visit 1 Categorical	
Sex	Percentage (n)
Female	40.53 (107)
Male	59.47 (157)
Race	
American Indian/Alaskan Native	1.14 (3)
Asian	2.27 (6)
Native Hawaiian or Other Pacific Islander	1.14 (3)
Black or African American	10.23 (27)
White	69.70 (184)
More than one race	12.12 (32)
Unknown/not reported	3.41 (9)
Ethnicity	
Hispanic/Latinx	18.18 (48)
Not Hispanic/Latinx	77.65 (205)
Unknown/not reported	4.17 (11)
Diagnosis	
Idiopathic/other intellectual disability	33.33 (88)
Fragile X syndrome	31.06 (82)
Down syndrome	35.61 (94)

**Table 2:** Descriptive statistics for study variables (Visit 1)

	Mean	SD	Missing (n)
Chronological age (years)	15.52	5.17	0
SB5 Full Scale mental age* (years)	4.83*	2.12*	4
SB5 Full Scale deviation IQ	53.64	16.23	2
SB5 Nonverbal deviation IQ	55.62	15.16	1
SB5 Verbal deviation IQ	51.60	19.16	2
Vineland-3 ABC	52.60	17.01	16
			**Percent Valid**
NIHTB-CB DCCS ^1^	65.60	22.19	60.4(n = 160)
NIHTB-CB FICA ^1^	66.29	24.02	77.7(n = 206)
NIHTB-CB PVT ^2^	67.82	13.40	97.0(n = 257)
NIHTB-CB PSM ^1^	84.50	16.09	89.8(n = 238)
NIHTB-CB PCPS ^1^	70.63	20.62	78.1(n = 207)
NIHTB-CB ORRT ^2^	74.51	14.65	94.0(n = 249)
NIHTB-CB LSWM ^1^	63.99	15.08	54.7(n = 145)

* Non-normal distribution, median and IQR are reported.

1 indicates NIHTB-CB Fluid Composite subtest

2 indicates NIHTB-CB Crystalized composite subtest.

DCCS = Dimensional change card sorting; FICA = Flanker inhibitory control and attention; PVT = Picture vocabulary test; PSM = Picture sequence memory; PCPS = Pattern comparison processing speed; ORRT = Oral reading recognition test; and LSWM = List sorting working memory.

**Table 3:** Model fit statistic summary

Model	Cog	AB	Chi-sq	Scaled Chi-Sq	df	CFI	TLI	RMSEA
Model 1	Crystallized	Comm	241.92	243.50	57	0.899	0.845	0.124
Model 2	Fluid	Comm	300.48	309.24	150	0.909	0.887	0.079
Model 3	**Crystallized**	**DLS**	**128.22**	**128.86**	**57**	**0.964**	**0.945**	**0.075**
Model 4	**Fluid**	**DLS**	**243.23**	**250.42**	**150**	**0.941**	**0.927**	**0.067**
Model 5	**Crystallized**	**Soc**	**127.68**	**116.59**	**57**	**0.954**	**0.930**	**0.073**
Model 6	Fluid	Soc	423.65	435.87	150	0.782	0.732	0.115
Model A	Full	Comm	590.01	613.48	232	0.857	0.832	0.099
Model B	**Full**	**DLS**	**416.21**	**429.72**	**232**	**0.924**	**0.911**	**0.074**
Model C	**Full**	**Soc**	**406.70**	**417.30**	**232**	**0.899**	**0.882**	**0.078**
Full	Full	Full	1393.46	1372.52	573	0.850	0.836	0.085
Model 1.1	**Crystallized**	**Comm w/o written**	**81.59**	**84.58**	**34**	**0.960**	**0.928**	**0.081**
Model 2.1	**Fluid**	**Comm w/o written**	**201.57**	**209.97**	**115**	**0.923**	**0.901**	**0.073**

*Note*: Shaded models determined to have adequate fit. Goodness of fit according to Little (2013):

RMSEA: <0.01 = great, 0.05 – 0.01 = good, 0.08 – 0.05 = acceptable, 0.10 – 0.08 = mediocre, > 0.10 = poor

CFI: >0.99 = great, 0.95–0.99 = good, 0.90–0.95 = acceptable, 0.85–0.90 = mediocre, < 0.85 = poor

TLI: >0.99 = great, 0.95–0.99 = good, 0.90–0.95 = acceptable, 0.85–0.90 = mediocre, < 0.85 = poor

**Table 4.** Correlation of latent change (ΔAB and ΔCOG)

Model	Cog	AB		Estimate	SE	*p*
Model 1	Crystallized	Comm	0.496	6.21	2.71	.004
Model 2	Fluid	Comm	0.250	3.14	5.34	.557
Model 3	Crystallized	DLS	0.471	16.92	6.32	0.007**
Model 4	Fluid	DLS	−0.106	−1.76	6.985	.801
Model 5	Crystallized	Soc	0.230	10.34	7.38	0.161
Model 6	Fluid	Soc	0.979	3545.33	6.79	< .001***
Model A	Full	Comm	0.539	6.31	3.20	.049*
Model B	Full	DLS	0.462	10.30	4.99	.039*
Model C	Full	Soc	0.322	7.49	4.70	.111
Full	Full	Full	0.462	8.58	4.15	.039*
Model 1.1	Crystallized	Comm	0.447	12.44	7.47	.096
Model 2.1	Fluid	Comm	0.395	5.79	6.80	.394

*Note*: Significant paths are denoted by

* p < 0.05

** p < 0.01

*** p< 0.001.

Shaded rows denote models with good fit.

**Table 5:** Cross-domain coupling of cognition at Visit 1 and ΔAB and ΔCOG

Model	Predictor	Outcome	β	Estimate	SE	*p*
Model 1	Crystalized Cognition	ΔAB COMM	0.39	0.180	0.960	.062
		ΔCOG CRYS	0.26	−0.225	0.072	.002**
Model 2	Fluid Cognition	ΔAB COMM	0.32	0.114	0.047	.015*
		ΔCOG FLUID	0.33	0.120	0.099	.223
Model 3	Crystalized Cognition	ΔAB DLS	0.28	0.217	0.117	.063
		ΔCOG CRYS	−0.06	−0.027	0.077	.728
Model 4	Fluid Cognition	ΔAB DLS	0.49	0.272	0.066	< .001***
		ΔCOG FLUID	0.43	0.153	0.093	.100
Model 5	Crystalized Cognition	ΔAB SOC	0.23	0.176	0.092	.055
		ΔCOG CRYS	−0.05	−0.024	0.049	.622
Model 6	Fluid Cognition	ΔAB SOC	−47.5	−83.4	23.90	< .001***
		ΔCOG FLUID	−235	−222	63.46	< .001***
Model A	Cognition Composite	ΔAB COMM	.46	0.215	0.117	.065
		ΔCOG FULL	−0.01	−0.002	−.102	.987
Model B	Cognition Composite	ΔAB DLS	0.38	0.323	0.113	.004**
		ΔCOG FULL	0.24	0.086	0.069	.218
Model C	Cognition Composite	ΔAB SOC	.312	0.295	0.096	.002**
		ΔCOG FULL	.22	0.080	0.052	.123
Full	Cognition Composite	ΔAB FULL	0.35	0.237	0.088	.007**
		ΔCOG FULL	0.21	0.074	0.065	.257
Model 1.1	Crystalized Cognition	ΔAB COMM	0.31	0.158	0.086	.066
		ΔCOG CRYS	−.09	−0.041	0.073	.581
Model 2.1	Fluid Cognition	ΔAB COMM	0.33	0.138	0.047	.003**
		ΔCOG FLUID	0.34	0.124	0.084	.138

*Note*: Significant paths are denoted by

* p < 0.05

** p < 0.01

*** p < 0.001.

Shaded rows denote models with good fit.

**Table 6:** Cross-domain coupling of AB at Visit 1 and ΔAB and ΔCOG

Model	Predictor	Outcome	β	Estimate	SE	*p*
Model 1	AB COMM	ΔAB COMM	−0.73	−0.433	0.127	.001**
		ΔCOG CRYS	0.55	0.298	0.095	.002**
Model 2	AB COMM	ΔAB COMM	−0.46	−0.298	0.107	.005**
		ΔCOG FLUID	0.14	0.091	0.169	.590
Model 3	AB DLS	ΔAB DLS	−0.58	−0.436	0.122	< .001***
		ΔCOG CRYS	0.03	0.014	0.069	.835
Model 4	AB DLS	ΔAB DLS	−0.70	−0.527	0.107	< .001***
		ΔCOG FLUID	−0.06	−0.031	0.126	.806
Model 5	AB SOC	ΔAB SOC	−0.54	−0.413	0.094	< .001***
		ΔCOG CRYS	−0.00	−0.001	0.045	.989
Model 6	AB SOC	ΔAB SOC	32.9	24.17	14.19	.089
		ΔCOG FLUID	165	65.34	37.54	.082
Model A	AB COMM	ΔAB COMM	−0.73	−0.440	−0.175	.012*
		ΔCOG FULL	0.26	0.122	0.128	.338
Model B	AB DLS	ΔAB DLS	−0.65	−0.489	0.113	< .001***
		ΔCOG FULL	−0.04	−0.013	−0.058	.825
Model C	AB DLS	ΔAB SOC	−0.57	−0.432	0.091	< .001***
		ΔCOG FULL	−0.00	−0.000	0.033	.996
Full	AB	ΔAB FULL	−0.53	−0.394	0.094	< .001***
		ΔCOG FULL	0.02	0.006	0.063	.920
Model 1.1	AB COMM w/o Written Comm.	ΔAB COMM	−.36	−0.277	0.135	.040*
		ΔCOG CRYS	0.10	0.063	0.114	.582
Model 2.1	AB COMM w/o Written Comm.	ΔAB COMM	−0.34	−0.267	0.142	.061
		ΔCOG FLUID	0.15	0.104	0.142	.465

*Note*: Significant paths are denoted by

* p < 0.05

** p < 0.01

*** p < 0.001.

Shaded rows denote models with good fit.

## Data Availability

Data are available from the NIMH Data Archive (nda.nih. gov/)—ID C3738.

## Abbreviations

IDD: intellectual and developmental disability
DSM: Diagnostic and Statistical Manual of Mental Disorders
NIHTB-CB: National Institutes of Health Toolbox Cognition Battery
VABS-3: Vineland Adaptive Behavior Scales, Third Edition
BLCS: bivariate latent change score
DLS: Daily Living Skills
FXS: fragile X syndrome
DS: Down syndrome
WS: Williams syndrome
FDA: Federal Drug Administration
SB5: Stanford Binet Intelligence Scales, Fifth Edition
FICA: Flanker Inhibitory Control and Attention
DCCS: Dimensional Change Card Sort
PCPS: Picture Comparison Processing Speed
LSWM: List Sorting Working Memory
PSM: Picture Sequence Memory
PV: Picture Vocabulary
ORR: Oral Reading Recognition
USS: unadjusted standard score
AB: adaptive behavior
CFI: comparative fit index
RMSEA: root mean square error of approximation
TLI: Tucker-Lewis index
IQR: inter-quartile range
COG: cognition
COA: clinical outcome assessment
GSV: growth scale value
ABC: Adaptive Behavior Composite
NBT: National Institutes of Health Baby Toolbox
